# Complete mitochondrial genome of *Polyopes lancifolius* and comparison with related species in Halymeniales (Rhodophyta)

**DOI:** 10.1080/23802359.2021.1908866

**Published:** 2021-04-08

**Authors:** Su Yeon Kim, Chung Hyun Cho, Eun Chan Yang, Hwan Su Yoon, Myung Sook Kim

**Affiliations:** a Korea Inter-University Institute of Ocean Science, Pukyong National University, Busan, Republic of Korea; b Department of Biological science, Sungkyunkwan University, Suwon, Republic of Korea; c Marine Ecosystem Research Center, Korea Institute of Ocean Science & Technology, Busan, Republic of Korea; d Department of Ocean Science, University of Science and Technology, Daejeon, Republic of Korea; e Department of Biology & Research Institute of Basic Sciences, Jeju National University, Jeju, Republic of Korea

**Keywords:** *cox*1 intron Halymeniales, mitochondrial genome, *Polyopes lancifolius*, Rhodophyta

## Abstract

*Polyopes lancifolius* is a species of Halymeniales, the fifth species-rich order within Rhodophyta. Using next-generation sequencing techniques, we recovered the complete mitochondrial genome of *P. lancifolius*, i.e. total 26,142 bp in length with 31% GC contents. A total of 49 functional genes were annotated, including 24 protein-coding, 23 transfer RNA, and 2 ribosomal RNA genes. The gene content and synteny have been highly congruent to those of the other halymenialean species, such as *Grateloupia taiwanensis, G. filicina*, and *Grateloupia angusta*. Interestingly, the *cox*1 intron and intronic Open Reading Frame (ORF) are absent in *P. lancifolius*, that are existed in the other three halymenialean species.

*Polyopes lancifolius* (Harvey) Kawaguchi & Wang is one of the introduced species in Europe originated from the northwest Pacific region (Mineur et al. 2010). This species is a member of Halymeniales, which is the fifth species-rich order (359 spp) within Rhodophyta (Guiry and Guiry 2020). However, only three mitochondrial genomes have been published in the order Halymeniales, i.e. *Grateloupia angusta* (KC875853; Kim et al. 2014), *Grateloupia taiwanensis* (KM999231; DePriest et al. 2014), and *Grateloupia filicina* (MG598532; Li et al. 2018). In this study, we sequenced and analyzed the complete mitochondrial genome of *P. lancifolius,* which is the first mitochondrial genome of the genus.

The specimens were collected on 25 January 2013 from Ganggu, Korea (36°21′29.6″N, 129°23′30.8″ E) and identified by *rbc*L phylogeny from the other halymenialean species. The voucher specimen (SKKU51) was deposited in Sungkyunkwan University (contact person: Su Yeon Kim, sykimcnu@gmail.com). Total genomic DNA was extracted using LaboPass^™^ Tissue Genomic DNA Isolation Kit Mini (Hokkaido System Science Co., Ltd., Sapporo, Japan) following manufacturer’s instruction. Ion Torrent PGM (Thermo Fisher Scientific, Waltham, MA) was applied for DNA sequencing using Ion PGM Template OT2 200 Kit and PGM Sequencing OT2 200 Kit. Total reads from genome data were assembled using CLC *de novo* assembler implemented in CLC Genomics Workbench version 6.5.1 (Aarhus, Denmark) (https://digitalinsights.qiagen.com). Candidate mitochondrial sequences were sorted from assembled contigs by comparing with reference halymenialean mitochondrial genes. The CDS and RNA genes were manually annotated by BLAST search using the NCBI nucleotide and protein database (nt, nr) and tRNA genes were predicted by tRNA-scanSE (Lowe and Chan 2016) and ARAGORN (Laslett and Canback 2004). After the annotation, the complete genome of *P*. *lancifolius* was submitted to GenBank (accession number MW292567).

The complete mitochondrial genome of *P. lancifolius* is 26,142 bp in length with 31% GC contents. The overall nucleotide composition is: 9434 bp of A (36.1%), 8597 bp of T (32.9%), 3968 bp of C (15.2%), and 4133 bp of G (15.8%). The genome is comprised 49 genes, including 24 protein-coding, 23 transfer RNA, and 2 ribosomal RNA genes. The newly constructed mitochondrial genome has a highly conserved gene synteny with three other halymenialean species, such as *G. taiwanensis, G. filicina*, and *G. angusta*. However, genomic differences were identified in the absence of *cox*1 intron and the number of tRNA genes. All species within the order Halymeniales have a trnI (tRNA Ile) intron, whereas *cox*1 intron and intronic Open Reading Frame (ORF) were found only in the three *Grateloupia* species, except for *P. lancifolius*. The total number of tRNAs of *P. lancifolius* is 23, which is higher than that of *G. angusta* (19) and lower than *G. taiwanensis* (24) and *G. filicin*a (24).

Total six mitochondrial genomes were selected to infer the phylogenetic relationships of *P. lancefolius* within the order Halymeniales. *Rhodymenia pseudopalmata* (KC875852) and *Sebdenia flabellata* (KJ398164), which are known as the sister orders of Halymeniales (Lee and Kim 2019; Yang et al. 2016), were used as outgroups. The maximum likelihood (ML) method was used to infer the phylogenetic relationship, and the tree was constructed using RAxML program (Stamatakis 2006). The best ML tree based on 24 CDS combined data (total 17,572 bp) fully supported (100% ML bootstrap support) the monophyly of the order Halymeniales and interspecies relationships within the order (Figure 1). Considering the current taxonomical classification and our mitochondrial genome phylogeny, only three *Grateloupia* species have a *cox*1 intron and intronic ORF within the order Halymeniales (Figure 1). The complete mitochondrial genome would contribute to understandings on the *cox*1 intron evolution in Rhodophyta.

**Figure 1.**
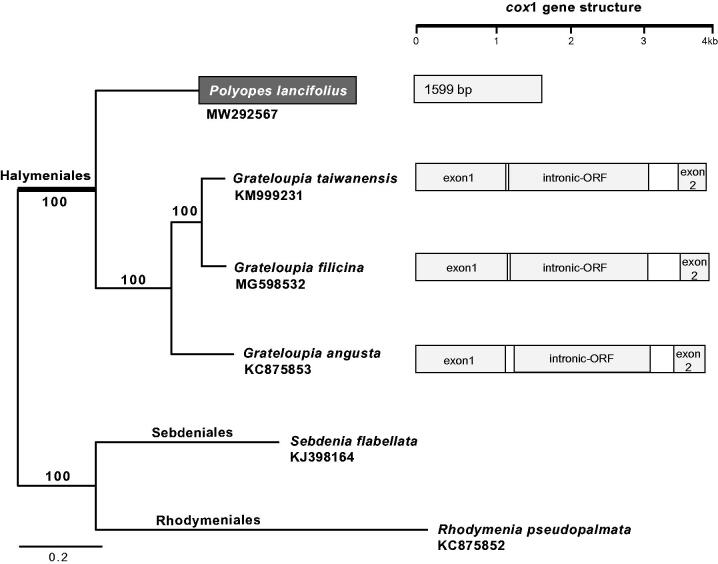
The maximum likelihood (ML) phylogeny of the order Halymeniales based on 24 combined CDS data (total 17,572 bp) of six species with *cox*1 gene structure. The monophyly of clade supported by the ML bootstrap support values (1000 replications).

## Data Availability

The genome sequence data that support the findings of this study are openly available in GenBank of NCBI at (https://www.ncbi.nlm.nih.gov/) under the accession no. MW292567. The associated SRA and Bio-Sample numbers are PRJNA690127 and SAMN17244873, respectively.
